# Investigating multi-material hydrogel three-dimensional printing for *in vitro* representation of the neo-vasculature of solid tumours: a comprehensive mechanical analysis and assessment of nitric oxide release from human umbilical vein endothelial cells

**DOI:** 10.1098/rsos.230929

**Published:** 2023-08-16

**Authors:** Lisa Asciak, Lauren Gilmour, Jonathan A. Williams, Euan Foster, Lara Díaz-García, Christopher McCormick, James F. C. Windmill, Helen E. Mulvana, Joseph C. Jackson-Camargo, Roger Domingo-Roca

**Affiliations:** ^1^ Department of Electronic and Electrical Engineering, University of Strathclyde, Glasgow, UK; ^2^ Department of Biomedical Engineering, University of Strathclyde, Glasgow, UK

**Keywords:** neo-vasculature, three-dimensional printing, tissue-mimicking, animal replacement, nitric oxide, endothelial cells

## Abstract

Many solid tumours (e.g. sarcoma, carcinoma and lymphoma) form a disorganized neo-vasculature that initiates uncontrolled vessel formation to support tumour growth. The complexity of these environments poses a significant challenge for tumour medicine research. While animal models are commonly used to address some of these challenges, they are time-consuming and raise ethical concerns. *In vitro* microphysiological systems have been explored as an alternative, but their production typically requires multi-step lithographic processes that limit their production. In this work, a novel approach to rapidly develop multi-material tissue-mimicking, cell-compatible platforms able to represent the complexity of a solid tumour's neo-vasculature is investigated via stereolithography three-dimensional printing. To do so, a series of acrylate resins that yield covalently photo-cross-linked hydrogels with healthy and diseased mechano-acoustic tissue-mimicking properties are designed and characterized. The potential viability of these materials to displace animal testing in preclinical research is assessed by studying the morphology, actin expression, focal adhesions and nitric oxide release of human umbilical vein endothelial cells. These materials are exploited to produce a simplified multi-material three-dimensional printed model of the neo-vasculature of a solid tumour, demonstrating the potential of our approach to replicate the complexity of solid tumours *in vitro* without the need for animal testing.

## 1. Introduction

Biomedical research heavily relies on animal testing to better understand disease progression [1]. Despite the biological similarities between animals and humans, animal procedures come with a multitude of ethical concerns, long experimental durations and often result in poor reproducibility [2]. Attempts have been made to reduce animal testing by developing tissue-mimicking models that emulate *in vivo* physiological conditions [3,4], or employ bioinformatics to predict cancer-related mutations [5]. Production of microphysiological systems requires the development of well-characterized tissue-mimicking materials that can, indeed, replace animal procedures while faithfully replicating the intricacy of the microvasculature. Organoids can replicate the function of organs such that they can be used to investigate developmental biology, diseases (such as cancers) and therapies [6,7]. However, many organoids employ Matrigel as tissue-mimicking hydrogel, which comes from mice, has mechanical limitations, and influences the organoid's response to certain drugs [6,7]. Soft tissues are complex structures exhibiting dynamic mechanical behaviour beyond the static domain, in addition to anisotropy, and nonlinear characteristics [8,9]. Moreover, their acoustic properties—generally the speed of sound (*c*) and acoustic attenuation coefficient (*α*)—are fundamental for applications in biomedical ultrasound [10,11], which is arising as an exciting field for drug delivery. Hence, the development of healthy and diseased mechano-acoustic tissue-mimicking materials is crucial for advancing towards animal-free biomedical experimentation and replacing animal models with laboratory-produced platforms.

Three-dimensional printing has been investigated extensively to produce tissue-mimicking constructs [12,13]. Continuous advances in three-dimensional printing have led to the development of specialized materials for specific biotechnological applications. In tissue engineering, for instance, extrusion-based bioprinting is used to produce solid parts using a bioink in a layer-by-layer fashion [14]. There are several properties of these bioinks (e.g. viscosity and gelling behaviour) that must be optimized to achieve the desired outcomes in terms of part resolution, mechanical properties and cell viability [15–17]. Vat-photo-polymerization three-dimensional printing is becoming an alternative to tackle some of these drawbacks, since it produces parts quicker and at higher resolutions [18–20]. Typically, the materials used for vat three-dimensional printing are monomers functionalized with acrylate groups (whether entirely synthetic or semi-synthetic based on natural monomers [21]), and their viscosity is less critical for successful three-dimensional printing than in extrusion bioprinters. The multifactorial preparation process of photo-responsive hydrogels allows for fine-tuning of their properties to match the desired tissue characteristics (e.g. stiffness, strength and viscoelasticity). There are several strategies that can provide improved structural and mechanical properties, namely, controlling the degree of methacrylation, the hydrogel components (i.e. the type and concentration of photoinitiator and photoblocker, (PB)) and the three-dimensional printing parameters. Another effective strategy for fine-tuning the properties of three-dimensional printed hydrogels is the use of interpenetrating polymer networks (IPNs), which typically comprise two polymer chains intertwined on the molecular scale, therefore benefitting from the properties of multiple monomers and broadening the range of materials that can be used.

To fabricate functional tissues to replace animal testing, it is essential to produce materials that support cell attachment, proliferation and differentiation without altering cellular morphology and function. There has been a great deal of research focusing on the development of cell encapsulated hydrogels for tissue engineering applications [22–24]. Three-dimensional hydrogel environments provide a good platform for cell encapsulation because they mimic the natural extracellular matrix (ECM) of tissue, and provide suitable mechanical support for cellular adhesion, spread and migration within the matrix [25–27]. Nevertheless, in these environments, the cells are surrounded by the hydrogel material, and factors like pore size and porosity of the hydrogel can affect the diffusion of oxygen, nutrients and waste products, thereby influencing cell viability and behaviour [28,29]. Cells seeded on hydrogels, on the other hand, are typically exposed to the cell culture media and can freely exchange nutrients and waste products with it. This difference in nutrient and oxygen diffusion, together with the hydrogel's stiffness and chemical composition, can improve cell viability, proliferation, differentiation and function [13,30]. The constant release of nitric oxide (NO) by endothelial cells, for instance, has been shown to play a crucial role in promoting endothelial cell proliferation and vascularization in hydrogel-based systems [31,32]. Therefore, when designing three-dimensional printable biomaterials for microvascular tissue engineering, this factor should be considered in order to produce *in vitro* tissue-mimicking materials that replicate the endothelial function.

Cell attachment, spreading and migration within (or on) hydrogel matrices depend on the formation of focal adhesions (FAs) [33,34]—dynamic complexes that link the cytoskeleton to the ECM. The cytoskeleton, a network of protein filaments including actin, provides mechanical support and controls cell shape and motility [34,35]. The formation and turnover of FAs are regulated by various signalling pathways and mechanical cues, including substrate stiffness and topography, and play a crucial role in regulating intracellular signalling events while transmitting forces from the cytoskeleton to the ECM and vice versa [36,37]. Therefore, understanding the interplay between the cytoskeleton, FAs and the ECM is critical for designing hydrogel matrices that support cell behaviour in tissue engineering applications.

The objective of this work is to design and produce, via vat three-dimensional printing, a simplified multi-material representation of the neo-vasculature of a solid tumour with the aim to progress towards replacement of animal-based *in vivo* biomedical experimentation. To achieve this, a series of photo-responsive hydrogels were developed and investigated, focusing on their (i) microstructural, (ii) acoustic, (iii) elastic and viscoelastic mechanical properties, and (iv) cell compatibility. Human umbilical vein endothelial cells (HUVECs) were employed to determine whether or not the investigated hydrogels support cellular adhesion and growth, and how their composition impacts cytoskeletal actin expression and FAs. Moreover, the NO released by HUVECs was assessed at multiple time points to better understand ECM–cell interplay. Multi-material three-dimensional printability of these hydrogels was investigated by producing entangled microvascular topologies, and their fluidic interconnectivity was analysed in a simplified representation of a solid tumour's neo-vasculature.

## 2. Material and methods

### 2.1. Statistical analysis

All the datasets are presented as the means ± standard error of the mean (s.e.m.) unless otherwise specified. One-way analysis of variance (ANOVA) was performed for multiple comparisons and the Tukey mean comparison method was employed to establish significance between groups. Letters represent significance between datasets as follows: the highest value in each graph is associated the letter *a*. Groups with a same letter indicate no significant difference between them, different letters indicate significant differences (*p* < 0.05), and multiple letters indicate significant difference at the 5% level.

### 2.2. Resin preparation and three-dimensional printing

Gelatin methacryloyl (GelMA) was synthesized according to previous protocols [21] (details in the electronic supplementary material).

Single monomer networks for three-dimensional printing consisted of 10% w/v poly(ethylene) glycol diacrylate (PEGDA, Mn 700 Da), bisphenol-A ethoxylate dimethacrylate (BEMA, 1.5 kDa, EO/phenol 15) and GelMA in de-ionized water including 34 mM of LAP and tartrazine (acting as PB) concentrations of 0 mM, 0.56 mM and 1.31 mM. IPNs consisting of a 1 : 1 ratio between PEGDA:GelMA, and BEMA:GelMA were equivalently prepared from stock solutions. All materials were obtained from Merck (Germany).

A firmware-modified PRUSA SL1S three-dimensional printer equipped with a 405 nm LED panel (PRUSA, Czech Republic) was used to produce all hydrogels at 50 µm layer thickness. For multi-material three-dimensional printing, the three-dimensional printer was pre-programmed to stop at a desired layer and recover its vertical zero position. During this stoppage time, the three-dimensional printed part was carefully cleaned *in situ* with de-ionized water to remove excess and trapped resin. After cleaning, the resin tank was swapped with another that contained a different material, the three-dimensional printing settings were adjusted, and the print was resumed until completion. The multi-material part was then removed from the build block, cleaned in de-ionized water and cured in a UV chamber (PRUSA CW1S) for 10 min.

The hardware-modified three-dimensional printer allows us to define different regimes where different three-dimensional printing parameters can be used. This is an important feature since each resin has different photo-polymerization kinetics, and as such, each resin needs to be previously calibrated. This is done via the Beer–Lambert law (details in the electronic supplementary material), which provides the characteristic parameters of each resin. These parameters, which include the penetration depth, *h_a_*, and the cure depth, *C_d_*, enable determination of the exposure times for a given layer thickness.

### 2.3. Microstructural characterization: porosity, gel and pore size, and microchannel morphology

Three-dimensional printed hydrogel discs (8 mm diameter, 2 mm thickness, *n* = 5) were lyophilized (Labconco FreeZone) for 24 h. The porous structure of the hydrogels was then investigated using micro-computed tomography (µCT; Bruker Skyscan, 1172, Belgium) at 2.5 µm isotropic voxel size. All scans were performed using 50 kVp tube voltage, 100 µA tube current, 829 ms exposure time, 0.3° rotation step (for a total of 180°), with frame averaging set to 2, and without any metal filters. The acquired hydrogel datasets were reconstructed using Skyscan NRecon software (Bruker, Version 1.6.9.18). Prior to three-dimensional morphometric analysis, a task list was created in CTAn software (Bruker, Version 1.20.8) to automatically generate a volume of interest to serve as the total volume over which porosity parameters were calculated (details in the electronic supplementary material).

Channel pattern fidelity was similarly investigated via µCT. Prior to scanning, the three-dimensional printed channels were perfused with a lead-loaded resin (33.33 mg ml^−1^, 44 µm lead particles in PlasClear, ASIGA) and photo-cross-linked in a UV light chamber (ASIGA Flash UV chamber) for 5 min. After resin solidification, the sample was scanned at 24 µm isotropic voxel size, 80 kVp tube voltage, 100 µA tube current, 850 ms exposure time, 0.2° rotation step (for a total of 180°), frame averaging set to 2 and employing both aluminium and copper filter. The acquired data were reconstructed as previously described.

### 2.4. Acoustic characterization: speed of sound and acoustic attenuation coefficient

Three-dimensional printed hydrogel discs (20 mm in diameter, 2 mm thickness, *n* = 5) were stored in de-ionized water to ensure a fully swollen state before testing. An unfocused ultrasound transducer (Olympus, A315S) with a centre frequency of 2.25 MHz was used to determine the speed of sound (*c*) through the materials, and the attenuation coefficient (*α*) was measured at 0.5 MHz, 1 MHz, 2.25 MHz and 5 MHz for each hydrogel via pulse-echo measurement (details in the electronic supplementary material).

### 2.5. Swelling behaviour

Three-dimensional printed hydrogel discs (20 mm diameter, 2 mm thickness, *n* = 3) were lyophilized for 24 h, and posteriorly weighed to obtain their initial dry weights (*W_d_*). The freeze-dried samples were immersed in 1X phosphate buffered saline (PBS) and incubated at 37°C. The wet weight (*W_s_*) of the gels was obtained at different timepoints (30 min, 1 h, 2 h, 72 h and one week) and the swelling ratio (*Q*) was calculated according to equation (2.1):

2.1 $$Q=\frac{W_{s}-W_{d}}{W_{d}}.$$

### 2.6. Unconfined compression stress relaxation

Three-dimensional printed hydrogel discs (8 mm diameter, 4 mm thickness, *n* = 5) were subjected to unconfined, uniaxial compression tests using the MACH-1 (Biomomentum Inc., Canada) mechanical testing system equipped with a 1.5 N load cell. The samples were tested at room temperature using a flat, impermeable stainless-steel indenter (12.5 mm diameter) in a cylindrical chamber (37 mm diameter) filled with de-ionized water to exclude the effects of sample dehydration. The thickness (*h*) of each sample was measured using a pre-defined pipeline in MACH-1 (details in the electronic supplementary material). Prior to compression, the hydrogel was preloaded to an amplitude of 1% *h*, at a 0.4% s^−1^ deformation rate to ensure contact between plate and sample. Each sample was then compressed to 15% strain at 5% increments relative to *h*. After each 5% compression ramp, a 10 min hold phase was implemented to investigate the stress relaxation behaviour of the hydrogel. All the compression steps were performed at a deformation rate of 0.1% s^−1^ (relative to *h*). The elastic modulus (*E*) was calculated from the slope of the linear region of the stress–strain curve for each ramp.

### 2.7. Rheological characterization

Three-dimensional printed hydrogel discs (20 mm diameter, 2 mm thickness) were first subjected to a 0.1 N pre-compression to ensure contact between plate and sample. To determine the linear viscoelastic regime (LVER), oscillatory shear strain sweeps were performed at shear strains ranging from 0.01% to 100% at a constant frequency of 1 Hz (*n* = 3). Once the LVER was identified, a shear strain of 0.01% was used to perform frequency sweeps (*n* = 5) from 0.1 Hz to 10 Hz to investigate the dynamic viscoelastic behaviour of the gels. All measurements were performed at room temperature using a Kinexus Pro+ rotational rheometer (NETZCH, Germany) equipped with a 20 mm parallel plate geometry.

### 2.8. Cell culture, viability and fluorescence staining

HUVECs (PromoCell, Germany) were maintained in endothelial cell growth medium supplemented 2% v/v fetal bovine serum (Supplement Mix, PromoCell), 1% v/v penicillin-streptomycin and 1% v/v l-glutamine. HUVECs were cultured at 37°C and 5% CO_2_, and used for no more than five passages. Hydrogels for cell culture were filtered using 0.22 µm sterile syringe filters (GP, Fisher Scientific, UK) prior to gelation. Before cell seeding (10 000 cells cm^−2^), the samples were UV-sterilized for 2 h and incubated overnight in complete HUVEC growth medium. Cell viability was monitored over a 7-day period via phase contrast microscopy (Motic AE31, Germany) employing a 20× objective. Endpoint live–dead staining was performed on day 7 using propidium iodide (20 µg ml^−1^) and Calcein-AM (5 µM). Fluorescence imaging was performed on a ZOE Fluorescent Cell Imager with a 10× objective.

The cells on the hydrogels and tissue culture plastic (TCP) were fixed prior to nuclei, filamentous actin (F-actin) and FA fluorescence imaging (details in the electronic supplementary material). Fluorescence images were taken on day 7 of cell culture using a ZEISS Axio Imager Z1 (ZEISS, Germany) microscope equipped with a 20× lens. The exposure times were set to 50 ms, 250 ms and 800 ms for the filters used to image Hoechst 33342, phalloidin and vinculin, respectively. The fluorescence images were used for morphological, phalloidin and vinculin analyses via CellProfiler [38] (details in the electronic supplementary material).

### 2.9. Nitric oxide release

Samples for NO analysis were prepared by pipetting 500 µl of hydrogel solution into 35 mm Petri dishes (fully covering the base of the Petri dish, *n* = 3). The samples were then placed in the UV chamber for 3 min, and subsequently seeded with HUVECs (10 000 cells cm^−2^). Samples were kept in 1 ml of complete HUVEC media at 37°C and 5% CO_2_. Negative controls were analogously prepared without cells. On days 1, 3 and 7, the media from each sample was collected and centrifuged at 13G for 15 min, and 100 µl of supernatant was used for NO testing.

HUVEC NO release was measured using the Griess assay (Griess reagent assay kit, Merck; details in the electronic supplementary material), and quantified after Griess standard curve calibration. The results were normalized against the negative controls.

## 3. Results

### 3.1. Microporous structure of the three-dimensional printed hydrogels

**Figure 1.**
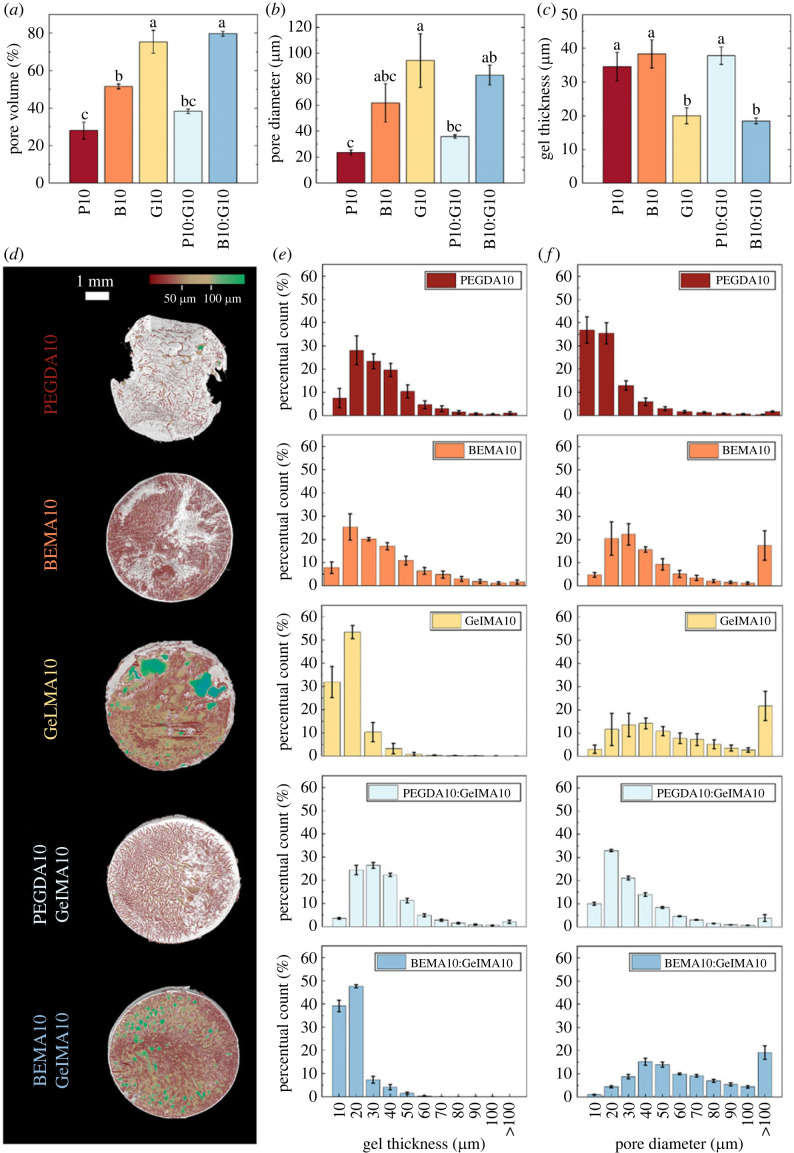
(*a*), (*b*) and (*c*) show, respectively, the average pore volume, pore diameter and gel thickness of each hydrogel formulation (*n* = 5 ± s.e.m.). Panel (*d*) Shows a representative µCT reconstruction of each hydrogel formulation. The white volume represents the gel (mesh), and the coloured volumes indicate the pore size distribution following the colour scale provided at the top of the figure. Panels (*e*) and (*f*) show the average hydrogel thickness (〈t_g_〉) and porosity (P) distributions, providing each hydrogel with overall microstructure signatures. Each representative image is accompanied by the average distributions of each group (*n* = 5 ± s.e.m.). Movies showing the internal structure of the micropores are provided in the electronic supplementary material.

The micropore structure revealed that PEGDA10 exhibits the lowest degree of porosity (28 ± 10%; figure 1*a*) and the lowest average pore diameter, 〈*d_p_*〉 (24 ± 4 µm; figure 1*b*), concomitantly leading to higher average gel thickness, 〈*t_g_*〉 (figure 1*c*). By contrast, GelMA10 exhibited a higher porosity value (73 ± 14%) and the largest 〈*d_p_*〉 (94 ± 46 µm), therefore showing thinner 〈*t_g_*〉. BEMA10 hydrogels presented values of porosity and 〈*d_p_*〉 (52 ± 3% and 62 ± 33 µm, respectively) between those measured in PEGDA10 and GelMA10. For the IPN hydrogels, PEGDA10:GelMA10 had porosity and 〈*d_p_*〉 values (38 ± 3% and 36 ± 3 µm, respectively) between those measured for PEGDA10 and GelMA10, while BEMA10:GelMA10 showed porosity and 〈*d_p_*〉 values (80 ± 3% and 83 ± 17 µm, respectively) similar to those measured in GelMA10 hydrogels. Figure 1*d* shows a representative µCT reconstruction of each hydrogel formulation, where the white volume represents the lyophilized hydrogel (mesh), and the coloured volumes indicate the pore size distribution following the provided colour scale. The gel thickness and porosity distributions (figure 1*e,f*) provide distinct microstructural signatures for each single-material hydrogel and illustrate how the individual materials contribute and combine in the IPN hydrogels.

### 3.2. Acoustic characterization and swelling behaviour

**Figure 2.**
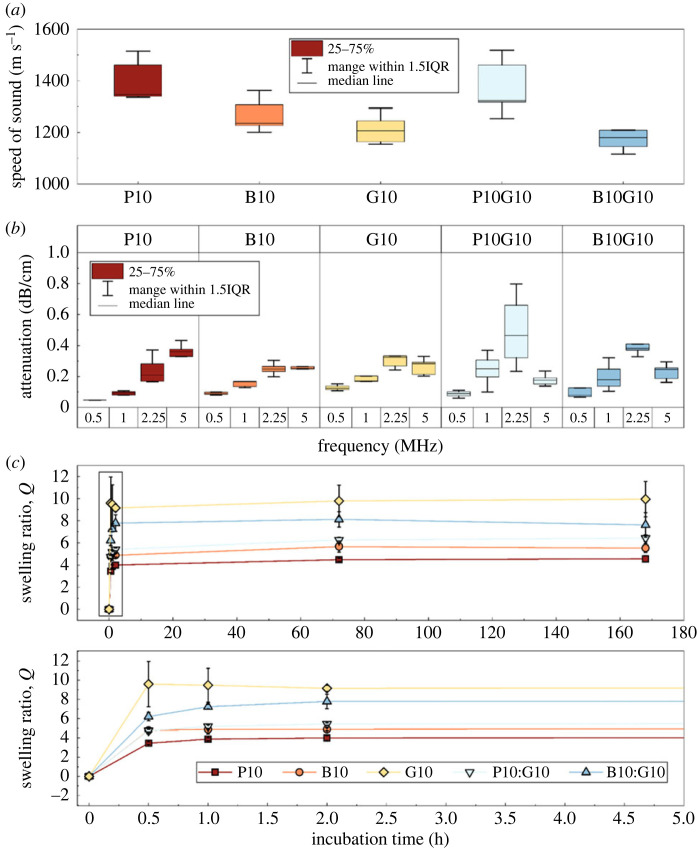
(*a*) Measured speed of sound of the five three-dimensional printed hydrogels measured at 2.25 MHz. (*b*) Acoustic attenuation coefficient of the five three-dimensional printed hydrogels at 0.5, 1, 2.25 and 5 MHz. (*c*) Swelling kinetics of the five hydrogel formulations. Bottom graph zooms in on the first 5 h. Error bars represent s.d. (*n* = 5 for speed of sound and acoustic attenuation, and *n* = 3 for swelling).

The measured average speed of sound, *c*, ranged from 1213 ± 52 m s^−1^ (GelMA10) to 1400 ± 74 m s^−1^ (PEGDA10) (figure 2*a*), with IPN *c* values being in between those measured for the single-network formulations. The range of measured acoustic attenuation coefficients (*α*) ranged from 0.049 ± 0.018 dB cm^−1^ (PEGDA10) to 0.49 ± 0.21 dB cm^−1^ (PEGDA10:GelMA10) at 0.5 MHz and 5 MHz, respectively (figure 2*b*). All hydrogels reached swelling equilibrium after 1 h immersion in 1x PBS (figure 2*c*), with GelMA10 and PEGDA10 showing, respectively, the greatest and lowest swelling ratios. IPN hydrogels reported swelling ratios in between those obtained in single-networked hydrogels.

### 3.3. Mechanical characterization

**Figure 3.**
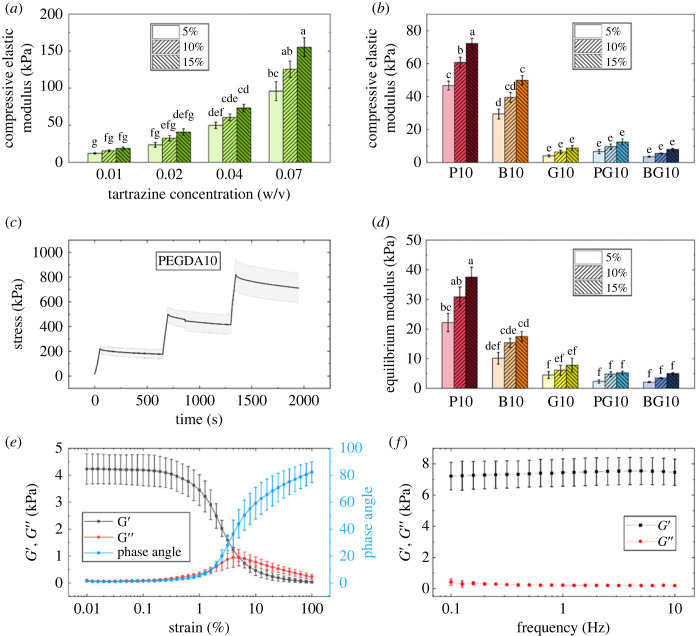
(*a*) Compressive elastic moduli dependency on photoblocker concentration at 5%, 10% and 15% compressive strains in PEGDA10. (*b*) Compressive elastic moduli of the five three-dimensional printed hydrogel formulations at 5%, 10% and 15% compressive strains. (*c*) Time-dependent evolution of the stress experienced by the PEGDA10 hydrogel formulation over a period of 2000 s at 5%, 10% and 15% strain loadings. The shaded area represents s.d. (*n* = 5). (*d*) Compressive equilibrium moduli values for each hydrogel formulation at 5%, 10% and 15% compression, calculated from the stress versus time plots. (*e*) Amplitude sweep showing the storage (*G*') and loss (*G*’’) moduli, and phase angle (*δ*) within the range of 0.01% to 100% strain of PEGDA10. Error bars represent s.d. (*n* = 3). (*f*) Frequency sweep showing the storage (*G*’) and loss (*G*’’) moduli within the range of 0.1–10 Hz of PEGDA10. For (*a*), (*b*), (*d*) and (*f*) error bars represent s.d. (*n* = 5). (*c*), (*e*) and (*f*) show representative plots of the tests for PEGDA10. The experimental data corresponding to the other hydrogel formulations can be found in the electronic supplementary material.

Unconfined compression stress relaxation tests revealed that PB concentration has a significant impact on hydrogel stiffness, leading to an eightfold increase in stiffness between PB concentrations of 0.19 mM and 1.31 mM (figure 3*a*). GelMA10 exhibited the lowest compressive stiffness values (4.02 ± 1.46 kPa at 5% compression; figure 3*b*), while PEGDA10 and BEMA10 had higher stiffness values (47 ± 6 kPa and 29 ± 7 kPa, respectively, at 5% compression; figure 3*b*).

All hydrogels displayed strain-dependent properties, revealing increasing compressive modulus with increasing strain (figure 3*b*). Strain-dependent stress relaxation was also measured for all hydrogels (figure 3*c*), confirming their viscoelastic behaviour. Interestingly, GelMA10 and, to some extent, BEMA10:GelMA10, showed increased stress during the hold phase (electronic supplementary material, figure S3). The resulting equilibrium moduli derived from a poroviscoelastic model are shown in figure 3*d*.

Rheological analysis revealed an LVER ranging from 0.01% to 0.1% shear strain. Within this region, the storage and loss moduli (*G*' and *G*’’, respectively) and the phase angle (tan(*δ*)) were constant for most hydrogels (figure 3*e*). Beyond the LVER, a decrease in *G*' could be observed, accompanied by an increase in *G*’’ and tan(*δ*). The *G*'-*G*’’ intersection point varies between 1% and 10% for BEMA10, PEGDA10 and PEGDA10:GelMA10, and between 10% and 100% for BEMA10:GelMA10 (electronic supplementary material, figure S4*g*). In GelMA10 hydrogels, no crossover was observed (electronic supplementary material, figure S4*e*), and despite showing the longest LVER, its low G' magnitude indicates weak bonding of the hydrogel structure. This phenomenon is improved via the formation of IPNs, where the *G*' magnitude is considerably higher than GelMA10 on its own. Frequency sweeps (figure 3*f*) showed that all hydrogels exhibit much higher *G*’ than *G*’’ within the 0.1–10 Hz range (electronic supplementary material, figure S4).

### 3.4. Multi-material three-dimensional printing of a simplified representation of a solid tumour's neo-vasculature

Following hydrogel morphological, acoustical and mechanical characterization, multi-material three-dimensional printing of a simplified representation of a solid tumour was demonstrated. Rather than precisely replicating the topology of a solid tumour, the possibility to produce three-dimensional multi-material channels was investigated with the aim to move away from conventional two-dimensional *in vitro* models that rely on single-material, in-plane channels. The characteristic parameters for each resin are shown in table 1.

**Table 1.** Characteristic parameters of each hydrogel formulation obtained in resin calibration using the PRUSA SL1S. (These parameters include penetration depth (*h_a_*) and cure depth (*C_d_*).)

hydrogel	*h_a_* (mm)	*C_d_* (mm)
PEGDA10	0.593	1.151
BEMA10	0.359	0.997
GelMA10	0.719	0.937
PEGDA10:GelMA10	0.504	1.601
BEMA10:GelMA10	0.741	1.455

**Figure 4.**
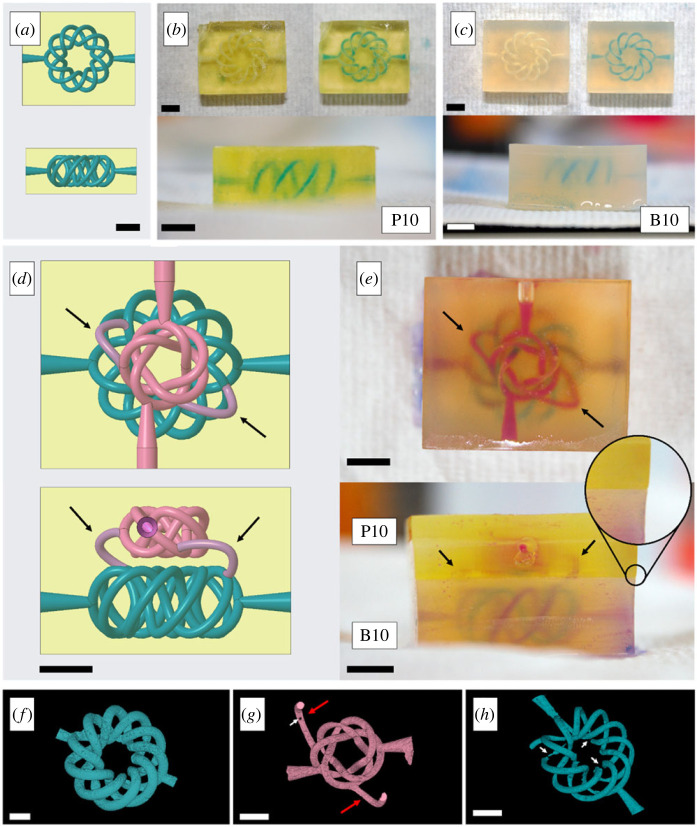
(*a*) CAD file of a (3,10) torus knot converted into a microvascular structure in PEGDA10 from the front and top views. (*b*) and (*c*) three-dimensional printed microvasculature in PEGDA10 and BEMA10, respectively, showing the empty and filled channels. (*d*) CAD file of two interconnected (3,10) and (3,5) torus knots (from the top and front views). Arrows indicate the parametric curves that connect the top torus knot (representing the solid tumour's vasculature) with the bottom torus knot (representing healthy vasculature). (*e*) Top and front views of the multi-material three-dimensional printed interconnected (3,10) and (3,5) torus knots. The arrows indicate the simplified replica of the neo-vasculature, where fluid migrates from the diseased-mimicking vasculature to the healthy-mimicking vasculature. The circle close-up shows the transition from BEMA10 to PEGDA10. Scale bars = 5 mm. (*f*) µCT reconstruction of the single-material three-dimensional printed (3,10) torus knot. Scale bar, 2.5 mm. (*g*) and (*h*) µCT reconstructions of the (3,5) and (3,10) torus knots, respectively. White arrows indicate small volumes of air trapped in the lead-loaded resin, and red arrows indicate the neo-vasculature. Scale bars = 1 mm.

To determine whether the tissue-mimicking materials supported independent three-dimensional printing of three-dimensional entangled networks, healthy (BEMA10) and diseased (PEGDA10) tissue-mimicking vasculature was produced (figure 4*a–c*). The three-dimensional printed topologies consisted of (3,10) torus knots, and their perfusion fidelity was shown with a blue dye (figure 4*b*,*c*). Since both PEGDA10 and BEMA10 supported three-dimensional printing of entangled three-dimensional structures, a (3,5) torus knot was added on top of the original (3,10) torus knot, and they were connected using two parametric curves aiming to reproduce, in a simple manner, the neo-vasculature of a solid tumour (figure 4*d*,*e*). The arrows indicate the channels connecting the two torus knots, and the close-up circle indicates the interface between PEGDA10 and BEMA10. Fluidic interconnection between the two topologies was demonstrated by independently circulating pink and blue dyes through the top and bottom channels, respectively. It was observed, qualitatively, that the pink dye migrated to the bottom torus knot via the simplified neo-vasculature (figure 4*e*), and mixed with the blue dye flowing within the healthy tissue-mimicking material (figure 4*e*).

Figure 4*f* shows the µCT reconstruction of the single-material three-dimensional printed (3,10) torus knot, confirming that three-dimensional entangled structures can be produced at large diameters (1.31 ± 0.13 mm on average). Figure 4*g*,*h* shows the µCT reconstructions of the interconnected (3,5) and (3,10) torus knots, respectively. The white arrows indicate small volumes of air trapped within the lead-loaded resin, and the red arrows indicate the parametric curves connecting the two topologies. The average diameter of the interconnected vascular system was measured to be 644 ± 150 µm, with a total volume of 164.3 µl.

### 3.5. Cell viability, actin expression and focal adhesions

**Figure 5.**
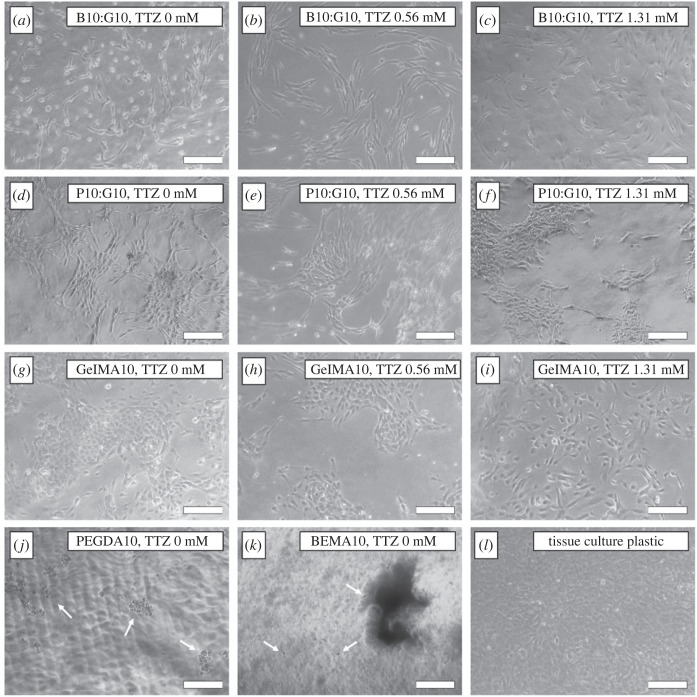
Representative phase contrast images of human umbilical vein endothelial cells (HUVECs) seeded on BEMA10:GelMA10 (*a*) without photoblocker (PB), (*b*) PB concentration 0.56 mM, and (*c*) PB concentration 1.31 mM. PEGDA10:GelMA10 (*d*) without PB, (*e*) PB concentration 0.56 mM (cells out of focus owing to the meniscus effect of the gel), and (*f*) PB concentration 1.31 mM. GelMA10 (*g*) without PB, (*h*) PB concentration 0.56 mM, and (*i*) PB concentration 1.31 mM. (*j*) PEGDA10, without PB (white arrows indicate rounded HUVECs), (*k*) BEMA10, without PB (white arrows indicate rounded HUVECs), and (*l*) tissue culture plastic without PB. Images taken on day 7. Scale bar, 100 µm.

Phase contrast images of hydrogel-seeded HUVECs (figure 5) revealed that PEGDA10 and BEMA10 did not support HUVEC growth or proliferation and, as such, they were excluded from any further cell-related investigations. Since PB concentration was observed to be a critical parameter in three-dimensional printing resolution and mechanical properties, its effect on cell activity at concentrations of 0 mM, 0.56 mM and 1.31 mM was investigated.

A limited number of HUVECs adhered to PEGDA10 and BEMA10 and displayed a rounded morphology rather than spreading and proliferating (figure 5*j*,*k*, white arrows). Live–dead assays revealed that these rounded cells remain viable throughout the 7-days period (details in the electronic supplementary material), suggesting that these hydrogels, despite not supporting cellular spreading or proliferation, are not cytotoxic to HUVECs. HUVECs were observed to adhere, spread and proliferate on all the other hydrogels. However, variations in HUVEC morphology were evident among the different hydrogels, with cellular elongation being the most prominent feature, and with BEMA10:GelMA10 substrates leading to more elongated HUVECs.

**Figure 6.**
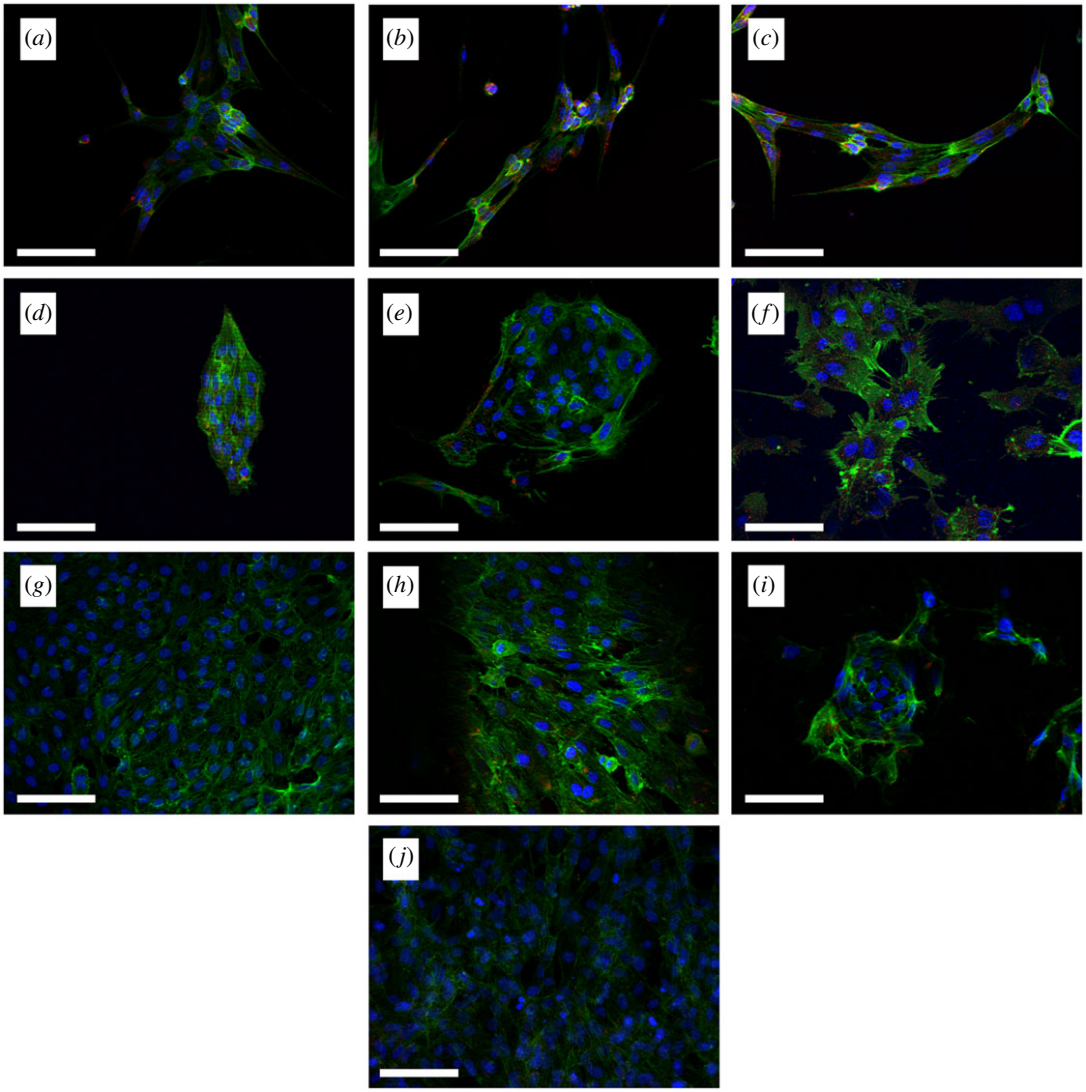
Merged fluorescence images of DAPI (blue), phalloidin (green) and vinculin (red) staining for human umbilical vein endothelial cells (HUVECs) seeded on BEMA10:GelMA10, (*a*) with photoblocker (PB) concentration of 0.19 mM, (*b*) PB concentration of 0.56 mM, and (*c*) PB concentration of 1.31 mM. PEGDA10:GelMA10, (*d*) with PB concentration of 0.19 mM, (*e*) PB concentration of 0.56 mM, and (*f*) PB concentration of 1.31 mM. GelMA10 (*g*) with PB concentration of 0.19 mM, (*h*) PB concentration of 0.56 mM, and (*i*) PB concentration of 1.31 mM. (*j*) tissue culture plastic (without PB). Images taken on day 7. Scale bar, 100 µm.

**Figure 7.**
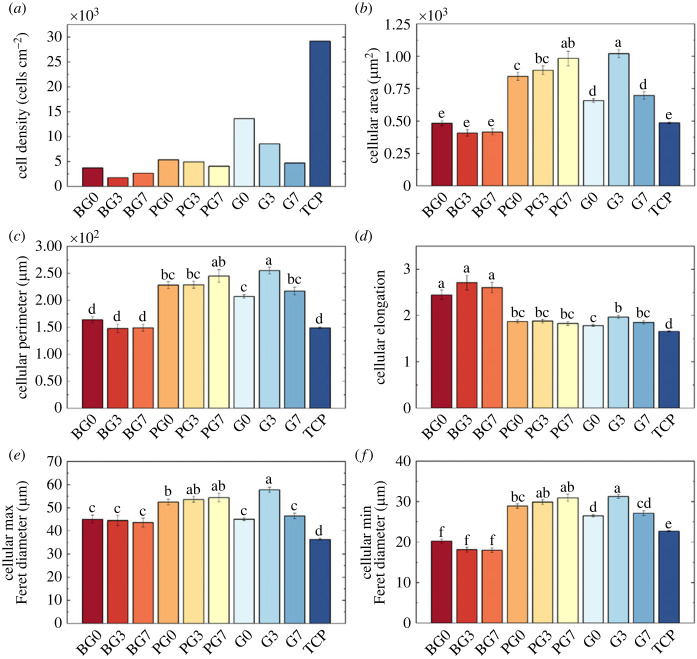
(*a*) Cell density, (*b*) total cellular area, (*c*) cellular perimeter, (*d*) cellular elongation, (*e*) cellular maximum Feret's diameter, and (*f*) cellular minimum Feret diameter for all the hydrogel formulations with increasing photoblocker concentration (with 0, 3 and 7 corresponding to 0 mM, 0.56 mM, and 1.31 mM, respectively). Data obtained on day 7 of cell culture. Lowercase letters indicate statistical significance described in the main text (*p*-values detailed in the electronic supplementary material). Error bars represent s.e.m.

Fluorescence microscopy (figure 6) enabled quantification of phalloidin and vinculin intensities, from which morphological information was obtained. Figure 6 shows an overlay of the Hoechst 33342 (blue), phalloidin (green) and vinculin (red) fluorescence images of HUVECs seeded on each hydrogel formulation on day 7 of cell culture. Increasing PB concentrations hindered cell proliferation on all hydrogels, with BEMA-cultured and GelMA-cultured HUVECs showing, respectively, the lowest and highest cell densities (figure 7*a*). Cellular area and perimeter were observed to clearly vary between HUVECs cultured on different hydrogels, with BEMA-cultured HUVECs revealing the most similar values to those observed on TCP (positive control), and with PEGDA-based hydrogels displaying significantly larger areas and perimeters than those observed on TCP (figure 7*b*,*c*). Despite showcasing the most similar areas to TCP, BEMA-cultured HUVECs were more elongated (up to 1.63 times more) than those measured on TCP, while the rest of the hydrogels yielded HUVECs that were similarly elongated to those measured on TCP. G0 provided the most similar readings (1.07 times TCP elongation). It was noted that the greatest elongation within each hydrogel subset corresponded to 0.56 mM PB. These elongational changes were confirmed by analysing the maximum and minimum Feret's diameters (figure 7*e*,*f*, respectively): BEMA-cultured HUVECs had lower and higher, respectively, minimum and maximum Feret's diameters than those measured on TCP-cultured HUVECs. On the other hand, HUVECs seeded on the other hydrogels showed similar variations of both minimum and maximum Feret's diameters, therefore leading to relatively constant elongation values. Further details on morphological analysis are shown in the electronic supplementary material.

**Figure 8.**
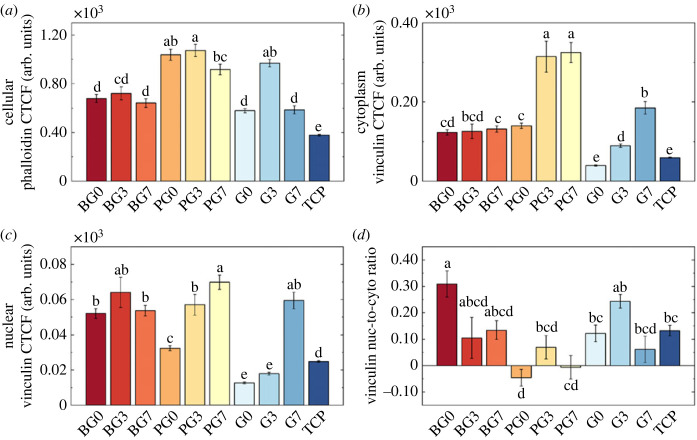
Corrected total cell fluorescence (CTCF) intensity for (*a*) cellular phalloidin, (*b*) vinculin present in the cytoplasm, (*c*) vinculin present in the nucleus, and (*d*) the ratio of vinculin present in the nucleus with respect to the amount of vinculin present in the cytoplasm, represented in a logarithmic scale. Positive values indicate higher amounts of vinculin in the nucleus, and negative values indicate more vinculin present in the cytoplasm. Data obtained on day 7 of cell culture. Lowercase letters indicate statistical significance as described in the main text (*p*-values detailed in the electronic supplementary material). Error bars represent s.e.m.

Phalloidin staining allowed visualization of F-actin and revealed that PEGDA-cultured HUVECs displayed the greatest F-actin expression (figure 8*a*). In all hydrogels, F-actin was overexpressed at PB concentrations of 0.56 mM, and downregulated at 1.31 mM, while vinculin was observed to be overexpressed at increasing PB concentrations (except for BG3; figure 8*b*,*c*). Cytoplasmic vinculin was significantly overexpressed on PEGDA-cultured HUVECs, while nuclear vinculin was measured to be of the same order of magnitude in BEMA- and PEGDA-cultured HUVECs. GelMA-cultured HUVECs showed similar vinculin expression to TCP-cultured HUVECs. Lastly, FA distribution along the cell was investigated (figure 8*d*) and it was observed that, on TCP, FAs are preferentially located on the nucleus. Similar distributions were observed in BG3, BG7, PG3, G0 and G7. BG0 and G3 displayed a nucleus-to-cytoplasm vinculin ratio three times higher than that observed on TCP-cultured HUVECs. PG7 showed the most uniform FA distribution, and PG0 had preferential FA distribution in the cytoplasm.

### 3.6. Nitric oxide release

**Figure 9.**
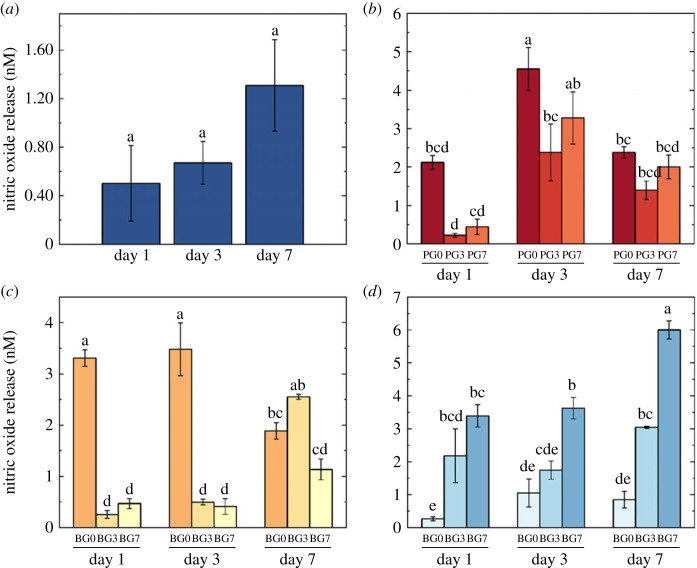
Nitric oxide (NO) release by human umbilical vein endothelial cells (HUVECs) on days 1, 3 and 7 of cell culture after normalization against the negative controls on (*a*) tissue culture plastic (TCP), (*b*) PEGDA10:BEMA10 hydrogels in the presence of 0 mM, 0.56 mM and 1.31 mM of photoblocker (named PG0, PG3 and PG7, respectively), (*c*) BEMA10:GelMA10 hydrogels in the presence of 0 mM, 0.56 mM and 1.31 mM of photoblocker (named BG0, BG3 and BG7, respectively), and (*d*) GelMA10 hydrogels in the presence of 0 mM, 0.56 mM and 1.31 mM of photoblocker (named G0, G3 and G7, respectively). Lowercase letters indicate statistical significance as described in the main text (*p*-values detailed in the electronic supplementary material). Error bars represent s.e.m.

Figure 9 shows the NO levels released by HUVECs on all the tested substrates. NO levels in HUVECs increased over 7 days in TCP, PEGDA, BEMA and GelMA cultures. In TCP-cultured HUVECs, NO production increased by 35% from days 1 to 3, followed by an increase of the 95.5% from days 3 to 7 (figure 9*a*). In PEGDA-cultured HUVECs (figure 9*b*), NO levels increased on day 3 compared to day 1 regardless of PB concentration, and decreased on day 7 (41.7% and 39.4% for PG3 and PG7, respectively). BEMA-cultured HUVECs (figure 9*c*) showed increasing NO concentrations over 7 days (BG3 and BG7), but BG0-cultured HUVECs produced similar NO levels on days 1 and 3, but decreased on day 7. All GelMA-cultured HUVECs (figure 9*d*) produced more NO over the 7-day period, with G7 producing the greatest increase (76.5%) between days 1 and 7.

## 4. Discussion

Understanding the morphology of hydrogels is fundamental to assess their suitability as tissue-mimicking materials, since their microstructure determines their overall mechanical behaviour [39]. Differences in microporous hydrogel structures may arise from the dissimilarities in molecular weight between monomers, along with their distinct molecular structure. These could dictate the way in which PEGDA and BEMA cross-link with GelMA. PEGDA cross-links through the attachment of free radicals to the end of the monomer chain by opening the carbon–carbon bonds. However, BEMA's phenol moieties might produce phenol radical derivatives upon exposure to UV light [40,41] that could potentially bind to GelMA's methacrylate groups (in addition to the growth occurring at the chain ends). In either case, with such a hypothesis requiring further investigation, the porosity results suggest that all hydrogels possess microporous structures suitable for replication of healthy and diseased tissue-mimicking microenvironments [42].

Animal replacement models must respond well to ultrasound, since its biomedical use provides diagnostic and treatment tools for a range of diseases, including solid tumours [43]. Both *c* and *α* have been well characterized for many healthy and diseased tissues [10,44], and are important parameters in tissue-mimicking platforms. On average, *c* values were measured to be slightly lower than those measured for most soft tissues [10], and lower than the expected lowest threshold (1480 m s^−1^, *c* of water). This is likely to occur owing to air being introduced into the samples during three-dimensional printing. Values of *α* corresponded well with those reported in the literature for soft tissue [10]. Swelling experiments revealed that hydrogels with higher water uptake had lower *c* values. This happens because highly swelled hydrogels have a lower volume of solid material, directly influencing wave velocity through it. One would be tempted to establish a direct correlation between the microstructure of the hydrogels and their ability to take up water. Nevertheless, swelling is a kinetic process that couples mass transport and mechanical deformation [45], and as such the mechanical behaviour of the mesh will also influence the hydrogel's ability to swell.

Each photo-responsive hydrogel is characterized by a series of intrinsic parameters that determine their printability. The penetration depth *h_a_* determines the material's sensitivity to changes in light energy; lower *h_a_* values indicate better printability when the material is subject to light energy variations, while higher *h_a_* values indicate, in general, faster printability but higher sensitivity of the material to variations in energy. The maximum cure depth, *C_d_*, is the practical maximum thickness that a certain material can be cured, indicating that thicker single layers will be unachievable. Formulation parameters such as photoinitiator and PB concentration determine these parameters, in addition to the intrinsic monomer properties of the components used, such as their molecular weight and functionality. BEMA10 and BEMA10:GelMA10 provide, respectively, the lowest and highest *h_a_* values. This means that while BEMA10:GelMA10 will be produced faster, BEMA10 is the most stable formulation to variations in light energy. All *C_d_* values were measured to be, roughly, above 1 mm, indicating that all hydrogels can be successfully three-dimensional printed at 50 µm. This value decreases with increasing PB concentration.

High concentrations of PB provide better three-dimensional printing resolutions but must be balanced with tissue-mimicking properties and cell viability. Higher PB concentrations lead to compressive moduli that exceed those exhibited by soft tissue. This happens because elevated PB concentrations require more time to reach gelation, therefore resulting in high cross-linking densities. Adjusting PB concentration in single-networked hydrogels would be enough to produce tissue-mimicking materials. Nevertheless, IPN production can enhance the range of tissue-mimicking properties that can be produced while facilitating GelMA production, which is critical for cell adhesion. All the formulations investigated here may be used as tissue-mimicking materials, since soft tissues have *E* values between 0.1 and 10 kPa [46–48], and cancerous tissue can reach 550 kPa depending on tumour type and subtype [49–51]. Moreover, biological materials display strain-dependent stiffness [52] under physiological loads, as it is the case in all hydrogels investigated herein. This increase in hydrogel stiffness may be attributed to water loss from the porous structure of the gel; at low strains, the hydrogels exhibit low stiffness but with increasing strain, the water starts to migrate off the polymeric network, leading to increased stiffness. This indicates that as strain increases, the materials exhibit a more viscoelastic response.

In purely viscoelastic materials, stress relaxation happens owing to the reconfiguration of polymer chains and short-range motion of water molecules, while poroelasticity is dominated by long-range fluid flow through the hydrogel's pores. In covalently cross-linked hydrogels—like the ones reported here—the cross-links cannot be easily broken and/or re-formed by an applied force, and therefore stress relaxation is primarily dominated by poroelasticity [53,54]. For hydrogels displaying increasing stress during the hold phase, it is believed that, following the hydrogel's re-submergence in water for the compression stress relaxation tests, swelling remains a dominating factor, therefore inducing pushing forces towards the indenter. This is reflected as an increase of stress over time.

The LVER region indicates the range of shear stress/strain that the hydrogels can withstand without any irreversible effects on the three-dimensional cross-linked network. Since *G*’’ is significantly lower than *G*' within this region, the hydrogels behave, predominantly, elastically. As shear strain increases, *G*' decreases while *G*’’ increases, and beyond the point where the two moduli intersect, the viscous properties start dominating over the elastic properties. This is reflected in the decomposition of the internal hydrogel structure, leading to a fluid-like behaviour. This phenomenon is further supported by the increasing phase shift angle (tan(*δ*)) and suggests predominantly elastic behaviour. Frequency-dependent viscoelasticity was also noticeable predominantly in GelMA10, which exhibited an increase in *G*' with frequency. This suggests that GelMA10 may be prone to structural changes above 3 Hz, demonstrating its weak mechanical behaviour. The elastic nature of GelMA10, in addition to its microporous structure, might aid its swelling ability, as opposed to a more rigid PEGDA10, further supporting the results from figure 2*c*.

Since PB concentration plays a fundamental role in hydrogel three-dimensional printing resolution and mechanical properties, its influence on cell characteristics was investigated. Phase contrast microscopy revealed that, similarly to what was observed in previous work [12], BEMA10 and PEGDA10 do not support HUVEC growth or proliferation. Instead, all GelMA-based formulations supported cell adhesion, growth and proliferation, and the morphology of HUVECs changed depending on the substrate's properties. Fluorescence imaging revealed that cellular densities decreased with increasing PB concentration. The differences observed between hydrogels without PB are associated with the hydrogel's chemical composition and mechanical properties. The former is important for cell adhesion since arginine–glycine–aspartate (RGD) peptide motifs (present in GelMA) must be available to interact with specific receptors on the surface of cellular integrin. In single-networked GelMA, all the methacrylate groups are cross-linked to each other, and the availability of RGD motifs is uniquely dictated by PB concentration, with high cross-linking densities reducing the accessibility to RGD ligands. When GelMA is cross-linked with another monomer, the availability of RGD motifs is further reduced since it also depends on the ratio between monomers. The mechanical properties of the hydrogel also influence stiffness-sensitive HUVEC-ECM mechanosensing and mechanotransduction [55–58], leading to decreased cell migration with increasing ECM stiffness [59]. In our case, stiffer hydrogels are obtained by increasing PB concentration, and lead to decreased cell densities. Moreover, tartrazine becomes cytotoxic at certain concentrations [60], and its diffusional kinetics [13] are dictated by hydrogel porosity (details in the electronic supplementary material), therefore influencing cell viability. All these parameters are hypothesized to also influence HUVEC morphology, although it is not possible to disentangle with absolute certainty their individual contributions. In general, cellular area increases with increasing ECM stiffness [61,62], as observed in PEGDA- and GelMA-cultured HUVECs (except for G3), although BEMA-cultured HUVECs have statistically the same area at increasing PB concentrations.

Phalloidin staining revealed overexpressed F-actin in PEGDA-cultured HUVECs. Several studies have investigated the correlation between F-actin expression, cell elongation and substrate stiffness [63–65], concluding that F-actin is overexpressed in elongated cells and stiffer substrates. Additionally, RGD availability could further influence phalloidin expression, since HUVECs would have more attachment points and experience higher forces within the cytoplasm. Interestingly, the more elongated cells within each hydrogel subset showed F-actin overexpression.

Vinculin has been identified as a critical indicator of cell–matrix interactions [66,67], and significant upregulation of vinculin gene expression has been detected on stiffer substrates [61]. Increased vinculin expression was measured with increasing PB concentrations in all hydrogel formulations (except for nuclear vinculin in BG3), hence confirming this trend. FA distribution within the cell did not seem to follow a clear trend. G0 (which showed the most similar morphology to TCP) produced statistically identical vinculin distribution throughout the cell than that measured in positive controls. These results suggest that FA strength may be influenced by substrate stiffness and RGD availability rather than HUVEC morphology, while FA distribution could depend on cellular morphology.

NO production in TCP-cultured HUVECs can be explained by the increase in amount of cells over the 7-day period. While this is also the case for GelMA-cultured HUVECs, the increased NO levels are attributed to HUVEC–RGD interactions. RGD motifs have the potential to enhance NO production in HUVECs by activating the endothelial oxide synthase (eNOS) enzyme signalling pathway [68–70]. Additionally, RGD motifs can enhance the expression of the vascular endothelial growth factor (VEGF) [71–73], leading to an overall increase in angiogenic activity, which at the same time is associated with elevated NO levels. Increased substrate stiffness has been associated with NO impairment [74], further supporting the differences in NO production between TCP- and GelMA-cultured HUVECs. Variations in NO production could arise from trapped tartrazine within the polymeric matrix (details in the electronic supplementary material). GelMA samples featured the highest degree of porosity and the largest pore size, leading to faster tartrazine release (47% and 25% release on day 7 for G3 and G7, respectively) than that observed in less porous hydrogels (3% and 10% tartrazine release for PEGDA and BEMA samples, respectively, on day 7). These results suggest that tartrazine concentrations above 1 mM could contribute to the disruption of HUVEC NO release. While these hypotheses are in accordance with our results, further investigation is required to determine if tartrazine interferes with VEGF production and/or inhibits the eNOS pathway. Furthermore, enhanced cell–cell communication and cell–ECM adhesion can lead to elevated levels of NO [68], which would also require further investigation.

In conclusion, we have demonstrated production of a series of three-dimensional printable, tissue-mimicking, cell-compatible materials. From these, healthy and unhealthy tissue-mimicking materials were identified and employed to produce a three-dimensional multi-material simplified version of a solid tumour's neo-vasculature, moving away from the most commonly used single-material, two-dimensional channel systems. HUVEC compatibility, morphology and NO release on these materials showed that it is possible to study F-actin and FA expression in addition to, potentially, eNOS signalling. While the latter would require further specialized experimentation, the results are highly promising for future development of a fully seeded three-dimensional printed platform where more specific biomedical questions can be investigated.

While this platform is still at a preliminary stage, the aim of our work was to open the doors towards more realistic microvascular platforms to replace *in vivo* animal experimentation, such that new therapies, treatments and cellular processes can be ethically investigated in animal-free platforms while still obtaining reliable results. This work provides insight into the physical and biological characteristics of some of the hydrogels that could be exploited to produce, cost- and time-efficient, tissue-mimicking materials able to provide a laboratory-based *in vitro* platform to replicate complex three-dimensional microvascular environments.

## Data Availability

The data are provided in the electronic supplementary material [75].
